# Early start of seasonal transmission and co-circulation of West Nile virus lineage 2 and a newly introduced lineage 1 strain, northern Italy, June 2022

**DOI:** 10.2807/1560-7917.ES.2022.27.29.2200548

**Published:** 2022-07-21

**Authors:** Luisa Barzon, Fabrizio Montarsi, Erika Quaranta, Isabella Monne, Monia Pacenti, Alice Michelutti, Federica Toniolo, Patrizia Danesi, Giulio Marchetti, Federica Gobbo, Alessandro Sinigaglia, Silvia Riccetti, Emanuela Dal Molin, Laura Favero, Francesca Russo, Gioia Capelli

**Affiliations:** 1 Department of Molecular Medicine, University of Padova, Padua, Italy; 2 Microbiology and Virology Unit, Padova University Hospital, Padua, Italy; 3 Istituto Zooprofilattico Sperimentale delle Venezie, Legnaro, Padua, Italy; 4 Direzione Prevenzione, Sicurezza Alimentare, Veterinaria, Regione del Veneto, Venice, Italy

**Keywords:** West Nile virus, surveillance, lineage, climate change, One Health, genome sequencing

## Abstract

In spring 2022, Europe faced an unprecedented heatwave, increasing the risk of West Nile virus (WNV) outbreaks. As early as 7 June 2022, WNV was detected in *Culex* mosquitoes in northern Italy, and – in the following days – in two blood donors, a patient with encephalitis, wild birds and additional mosquito pools. Genome sequencing demonstrated co-circulation of WNV lineage 2 and a newly introduced WNV lineage 1, which was discovered in the region in 2021.

In 2022, the start of West Nile virus (WNV) activity in Europe was demonstrated as early as the beginning of June in the Veneto region, north-eastern Italy. We describe here WNV infections detected in mosquitoes, birds, and humans up to 8 July 2022 in the region, where a newly introduced WNV lineage 1 (WNV-1) strain co-circulated with WNV lineage 2. Phylogenetic analysis of full genome sequences of WNV-1 and WNV-2 detected in 2021 and 2022 is also reported.

## Integrated West Nile virus surveillance in Italy

In Italy, the surveillance system for WNV infection, which also includes Usutu virus (USUV), is run by the Ministry of Health and is based on a ‘One Health’ approach; the system combines human surveillance with veterinary and entomological surveillance [1]. The aim of the integrated surveillance is to timely detect WNV activity and promptly activate proper response measures to prevent transmission to humans, as well as to identify areas at high risk for USUV disease. The surveillance is active year-round and enhanced during the transmission season, from the beginning of May to the end of November. According to the integrated surveillance plan, the first detection of WNV infection in either mosquitoes, birds, equids or humans triggers the initiation of preventive measures to secure the safety of substances of human origin (SoHO) [1].

In the Veneto region, laboratory diagnosis of WNV infection in humans and in animals is performed by the Regional Reference Laboratory at Padova University Hospital and by the Istituto Zooprofilattico Sperimentale delle Venezie, respectively, as previously reported [2,3]. 

## Detection of the first West Nile virus infections in June 2022

WNV activity started very early in Italy in 2022. Detection of the first WNV-positive *Culex* mosquito pool occurred on 7 June 2022 in the Vicenza province of the Veneto region. Whole genome sequencing (WGS) of the pool confirmed WNV lineage 2 (WNV-2) infection. This finding triggered the screening by WNV nucleic acid testing (NAT) of all SoHO donors who were resident in the Veneto region or who had stayed for at least one night in the region during the 28 days before donation. 

**Figure 1:**
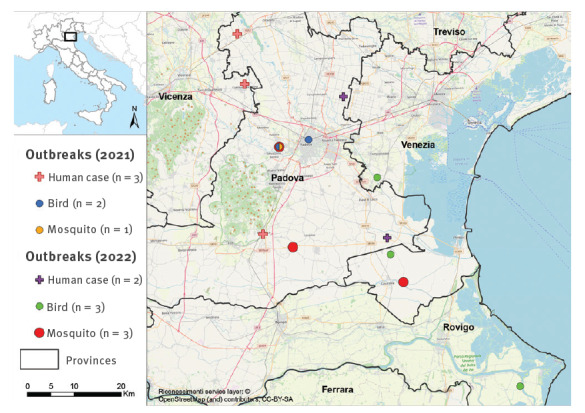
Sites of West Nile virus lineage 1 infections in humans, birds and mosquitoes, Veneto region, Italy, 2021–2022 (n = 14)

On 18 June 2022, a WNV NAT-positive blood donor was identified in Venice province. Two days after the donation, the donor reported flu-like symptoms that lasted for 3 days. WNV infection was also confirmed in a patient with encephalitis from Padua province, who developed symptoms on 28 June, and in another blood donor from Padua province (index donation on 8 July). In all three cases, WNV RNA was detected in blood and urine, and anti-WNV IgM antibodies in serum. Partial genome sequencing confirmed WNV-2 in the first blood donor and WNV-1 infection in the encephalitis case and in the second blood donor (shown in Figure 1).

The map shows the geographical sites in which WNV-1 was detected in 2021 compared to 2022. In 2021, WNV-1 was present mainly in Padua province (map, Italian: Padova) (n = 2 human cases, 2 bird cases and 1 site with WNV-positive mosquitoes) with only n = 1 human case in Vicenza province. In 2022, the presence of WNV-1 was confirmed in Padua province (n = 2 human cases, 2 sites with WNV-positive mosquitoes) and extended to Venice (map, Italian: Venezia) province (n = 2 birds and 1 site with positive mosquitoes) and Rovigo province (n = 1 bird). Simultaneous WNV-2 circulation was detected in Vicenza, Padua, Venice, Verona, and Rovigo provinces.

Veterinary surveillance, which is active year-round on dead birds, detected WNV RNA in organs from wild birds. WNV-1 was identified in a common raven (*Corvus* spp.) and an owl (*Athene noctua*) caught on 20 June and 27 June, respectively, in Venice province, as well as in a magpie (*Pica pica*) caught on 28 June in Rovigo province. WNV-2 was detected in organs of a hooded crow (*Corvus cornix*) and in a long-eared owl (*Asio otus*), both found dead in Rovigo Province on 10 June and 28 June, respectively. Bird samples were submitted to the National Reference Centre for Foreign Animal Diseases (CESME) in Teramo, Italy, for the diagnostic confirmation by molecular testing and sequencing. 

On 21 June and 5–7 July, another four *Culex* mosquito pools from a total of 843 pools collected during 2022 tested positive for WNV-1 in Venice (two positive pools at one site) and Padua (two positive pools at two sites) provinces (Figure 1), while a total of nine *Culex* mosquito pools tested positive for WNV-2 in Vicenza, Padua, Venice, Verona, and Rovigo provinces.

## Detection of West Nile virus lineage 1 in 2021

In 2021, 8 years after the last human cases of WNV-1 infection reported in Italy [4], WNV-1 infection was diagnosed in two patients with meningoencephalitis and one with fever from Padua and Vicenza provinces, with symptom onset on 2 August, 22 August, and 10 September, respectively (Figure 1). In the same year, WNV-2 was detected in a patient with neuroinvasive disease and in a blood donor; four other symptomatic West Nile cases were confirmed by demonstration of a high titre of WNV IgM antibodies and a positive neutralisation assay (data not shown).

Of 2,173 pools investigated within the surveillance programme, WNV was detected in 16 pools of *Culex pipiens*, one of *Ochlerotatus caspius* and one of *Aedes albopictus* collected between 27 July and 26 August 2021. WNV-2 was detected in all positive pools, except one WNV-1-positive pool with 93 *Cx. pipiens* mosquitoes collected in Padua province on 19 August (Figure 1). Further sampling at the same site for research purposes identified three additional WNV-1-positive mosquito pools collected between 19 and 27 August.

Veterinary surveillance on 1,934 birds detected WNV-1 in a dead Eurasian collared dove (*Streptopelia decaocto*) and in a common wood pigeon (*Columba palumbus*). Samples were collected on 11 and 22 October 2021, respectively, in Padua province (Figure 1). Another seven birds were found infected with WNV-2 in the provinces of Venice, Verona and Vicenza.

## West Nile virus genome sequencing and phylogenetic analysis

**Figure 2:**
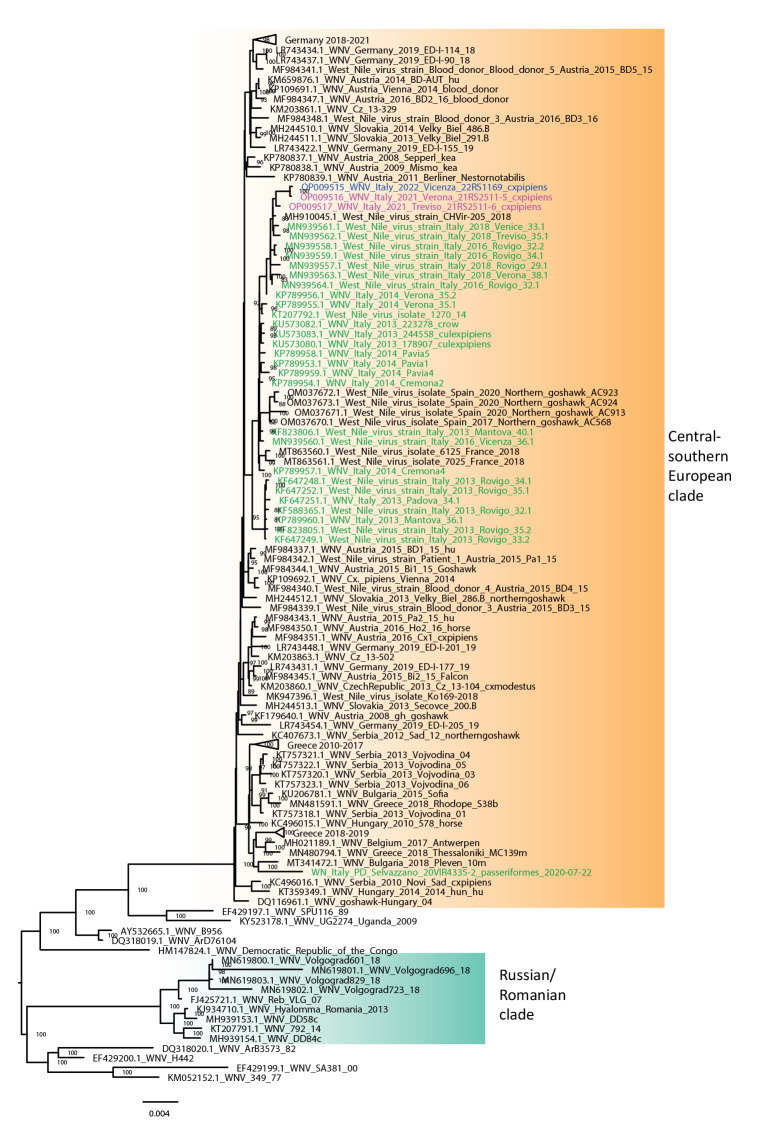
Phylogenetic tree of West Nile Virus lineage 2 strains, Veneto region, Italy, 2021–2022 (n = 3 sequences)

**Figure 3:**
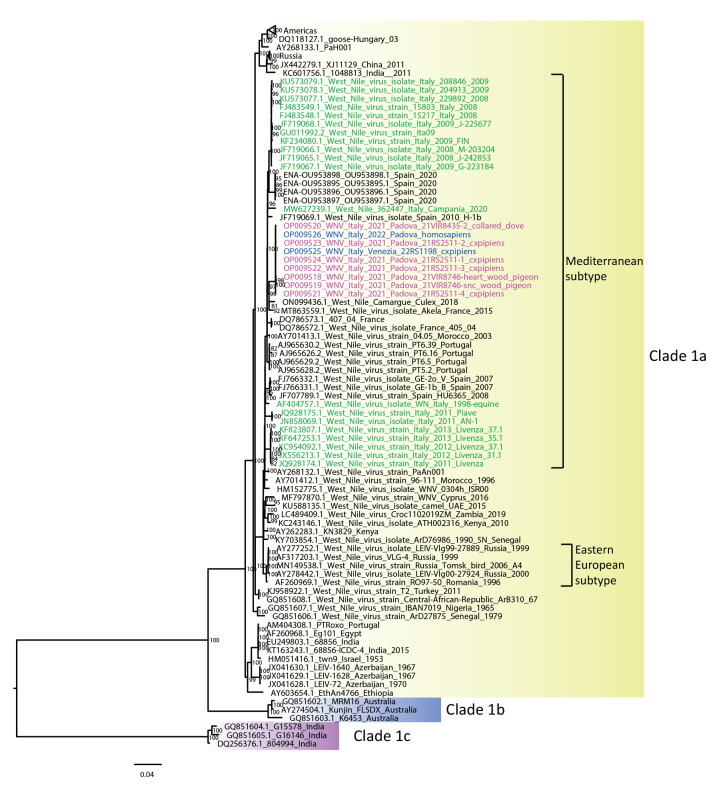
Phylogenetic analysis of West Nile Virus lineage 1 strains, Veneto region, Italy, 2021–2022 (n = 9 sequences)

Whole genome sequences were obtained for WNV-2 from two mosquito pools collected in 2021, while a partial sequence (3,792 nt) was obtained from one mosquito pool collected in June 2022. Phylogenetic analysis showed that these sequences clustered together within the central-southern European clade and presented an identity ≥ 99.6% with WNV-2 strains detected in the Veneto region since 2016 (Figure 2). Whole genome sequences were generated for WNV-1 strains detected in four mosquito pools and two wild birds in 2021 and one mosquito pool in 2022. The phylogenetic analysis revealed that the WNV-1 strains detected 2021 and 2022 were closely related to each other (99.9% sequence identity) and formed a distinct group within the western Mediterranean subtype of clade 1a [5]. A small sequence (182 nt) of the nonstructural protein 5 gene was obtained from the WNV-1 strains detected in humans in 2021 and 2022, which were identical to the sequences detected in mosquitoes and birds (Figure 3). Among published WNV sequences, the highest similarity (98.5% nucleotide sequence identity) was demonstrated with two viruses identified in southern France in 2015 and 2018 [6].

The maximum-likelihood phylogenetic tree was constructed with IQ-TREE (GTR + F + R3) and 1,000 bootstrap replicates. Ultrafast bootstrap supports higher than 80 are indicated next to the nodes. West Nile virus genomes sequences were generated by amplicon sequencing using an Illumina MiSeq platform.

The virus sequences analysed in this study are marked in fuchsia (n = 2 samples from *Culex pipiens* pools collected in 2021) and blue (n = 1 sample from a *Cx. pipiens* pool collected in 2022). Virus sequences collected in Italy before 2021 are displayed in green (n = 28). Background shading distinguishes two clades (Central-southern European clade in orange and Russian/Romania clade in green).

The maximum-likelihood phylogenetic tree was constructed with IQ-TREE (GTR + F + I + G4) and 1,000 bootstrap replicates. Ultrafast bootstrap supports higher than 80 are indicated next to the nodes. WNV genomes sequences were generated by amplicon sequencing using an Illumina MiSeq platform.

The viruses analysed in this study are marked in fuchsia (n = 4 samples from *Culex* mosquito pools and n = 2 wild birds collected in 2021) and blue (n = 1 human sample and n = 1 mosquito pool collected in 2022). Viruses collected in Italy before 2021 are displayed in green (n = 20). Background shading distinguishes three clades (clade 1a in yellow, clade 1b in blue and clade 1c in purple).

## Discussion

WNV is an emerging mosquito-borne pathogen that causes disease and death in humans, equids, and several bird species. During the last decade, WNV outbreaks have occurred across larger geographical areas in Europe, affecting an increasing number of countries [7], including Germany [8] and the Netherlands [9]. In 2018, the largest WNV outbreak ever recorded occurred in central and southern Europe, with over 2,000 symptomatic human cases – mostly in Italy [3,10] – and ca 9% case fatality rate [7]. 

The 2018 outbreak was sustained by WNV-2 of the central-southern European clade. WNV-2 was first discovered in Hungary in 2004 [11] and then became prominent with a large human outbreak in Greece in 2010 [12]. Thereafter, the virus spread to most European countries, including Italy, where it replaced WNV-1a strains [4,13,14]. We find here that the WNV-2 strain is still present in the country, as it was found in mosquitoes, birds, and human samples. In addition, we demonstrate the establishment of the newly introduced WNV-1a strain, which was identified for the first time in the region in 2021 and caused neuroinvasive disease and febrile illness in humans and infections in birds and mosquito vectors. Compared with 2021, in 2022 the virus spread to a larger geographical area affecting other provinces in the Veneto region. In addition, compared to the previous years, when WNV activity was generally detected in the Veneto region from mid-July with a peak in August [15], WNV seasonal transmission started earlier in 2022, probably as a consequence of favourable weather and ecological conditions. 

An artificial intelligence-based model that considered ecological and climate parameters as predictors of WNV outbreaks pinpointed that high spring temperatures had a pivotal role in determining the large WNV outbreaks that occurred in European countries in 2018 [16]. Another model based on publicly available data on the number of human infections recorded in Europe between 2011 and 2019 confirmed that spring temperature shapes WNV transmission in Europe [17]. In fact, low precipitation in winter and high temperatures during the spring are determinant in accelerating the WNV amplification cycle between birds and mosquitoes; this occurs through mechanisms that include enhanced replication of the virus in *Culex* mosquitoes [18] and increased growth rate of the mosquito population [19], which therefore increases biting and transmission rates.

An analysis of weather parameters that could have influenced WNV circulation during another large outbreak that occurred in northern Italy in 2013 showed that warmer and less rainy conditions were associated with a higher abundance of vector mosquitoes and with WNV circulation [15]. Such weather conditions, with very hot temperatures and drought, were recorded in 2022 in the Veneto region. According to weather data from the Regional Agency for Environmental Protection and Prevention of Veneto during the period from 1994 to 2022 [20,21], the winter season 2021/22 in the Veneto region had very warm temperatures and low precipitation; the period March–May 2022 was characterised by the lowest quantity of precipitation since 1997. In May 2022, the minimum temperatures were the highest since 1994, the maximum temperatures were the fourth highest in the 28-year period, while the daily averages were the second highest; precipitation levels were among the lowest since 1994 [20,21].

## Conclusions

The integrated ‘One Health’ approach for WNV surveillance detected the new WNV-1a strain first identified in 2021 again in 2022. The virus was reported simultaneously by the three sectors, i.e. in humans, birds and mosquitoes, in the same geographical area in north-eastern Italy, thus demonstrating the sensitivity of surveillance system. Systematic acquisition of virus genetic information in surveillance activities will be crucial to monitor the evolution of virus circulation and to achieve fundamental information on the epidemic and pathogenic potential of the new WNV-1a strain. In the context of unprecedented climate and environmental conditions in Europe, characterised by a prolonged heatwave and drought during spring [22], these findings suggest the importance of strengthening WNV surveillance and control measures, because of the risk of large human outbreaks [15,16].

## References

[1] Italian Ministry of Health. Piano Nazionale di prevenzione, sorveglianza e risposta alle Arbovirosi (PNA) 2020-2025. [National plan for prevention, surveillance and response to vector-borne viral diseases]. Rome: Italian Ministry of Health; 2019. Italian. Available from: https://www.salute.gov.it/imgs/C_17_pubblicazioni_2947_allegato.pdf

[2] Ravagnan S Montarsi F Cazzin S Porcellato E Russo F Palei M First report outside Eastern Europe of West Nile virus lineage 2 related to the Volgograd 2007 strain, northeastern Italy, 2014. Parasit Vectors. 2015;8(1):418. 10.1186/s13071-015-1031-y 26265490 PMC4534017

[3] Pacenti M Sinigaglia A Franchin E Pagni S Lavezzo E Montarsi F Human West Nile virus lineage 2 infection: epidemiological, clinical, and virological findings. Viruses. 2020;12(4):458. 10.3390/v12040458 32325716 PMC7232435

[4] Barzon L Pacenti M Franchin E Lavezzo E Masi G Squarzon L Whole genome sequencing and phylogenetic analysis of West Nile virus lineage 1 and lineage 2 from human cases of infection, Italy, August 2013. Euro Surveill. 2013;18(38):20591. 10.2807/1560-7917.ES2013.18.38.20591 24084339

[5] May FJ Davis CT Tesh RB Barrett AD . Phylogeography of West Nile virus: from the cradle of evolution in Africa to Eurasia, Australia, and the Americas. J Virol. 2011;85(6):2964-74. 10.1128/JVI.01963-10 21159871 PMC3067944

[6] Constant O Gil P Barthelemy J Bolloré K Foulongne V Desmetz C One Health surveillance of West Nile and Usutu viruses: a repeated cross-sectional study exploring seroprevalence and endemicity in Southern France, 2016 to 2020. Euro Surveill. 2022;27(25). 10.2807/1560-7917.ES.2022.27.25.2200068 35748300 PMC9229194

[7] Young JJ Haussig JM Aberle SW Pervanidou D Riccardo F Sekulić N Epidemiology of human West Nile virus infections in the European Union and European Union enlargement countries, 2010 to 2018. Euro Surveill. 2021;26(19):2001095. 10.2807/1560-7917.ES.2021.26.19.2001095 33988124 PMC8120798

[8] Pietsch C Michalski D Münch J Petros S Bergs S Trawinski H Autochthonous West Nile virus infection outbreak in humans, Leipzig, Germany, August to September 2020. Euro Surveill. 2020;25(46):2001786. 10.2807/1560-7917.ES.2020.25.46.2001786 33213686 PMC7678033

[9] Vlaskamp DR Thijsen SF Reimerink J Hilkens P Bouvy WH Bantjes SE First autochthonous human West Nile virus infections in the Netherlands, July to August 2020. Euro Surveill. 2020;25(46):2001904. 10.2807/1560-7917.ES.2020.25.46.2001904 33213687 PMC7678035

[10] Riccardo F Monaco F Bella A Savini G Russo F Cagarelli R An early start of West Nile virus seasonal transmission: the added value of One Heath surveillance in detecting early circulation and triggering timely response in Italy, June to July 2018. Euro Surveill. 2018;23(32):1800427. 10.2807/1560-7917.ES.2018.23.32.1800427 30107870 PMC6092914

[11] Bakonyi T Ivanics E Erdélyi K Ursu K Ferenczi E Weissenböck H Lineage 1 and 2 strains of encephalitic West Nile virus, central Europe. Emerg Infect Dis. 2006;12(4):618-23. 10.3201/eid1204.051379 16704810 PMC3294705

[12] Papa A Bakonyi T Xanthopoulou K Vázquez A Tenorio A Nowotny N . Genetic characterization of West Nile virus lineage 2, Greece, 2010. Emerg Infect Dis. 2011;17(5):920-2. 10.3201/eid1705.101759 21529413 PMC3321789

[13] Barzon L Franchin E Squarzon L Lavezzo E Toppo S Martello T Genome sequence analysis of the first human West Nile virus isolated in Italy in 2009. Euro Surveill. 2009;14(44):19384. 10.2807/ese.14.44.19384-en 19941775

[14] Barzon L Pacenti M Franchin E Squarzon L Lavezzo E Toppo S Novel West Nile virus lineage 1a full genome sequences from human cases of infection in north-eastern Italy, 2011. Clin Microbiol Infect. 2012;18(12):E541-4. 10.1111/1469-0691.12001 23004685

[15] Calzolari M Pautasso A Montarsi F Albieri A Bellini R Bonilauri P West Nile virus surveillance in 2013 via mosquito screening in northern Italy and the influence of weather on virus circulation. PLoS One. 2015;10(10):e0140915. 10.1371/journal.pone.0140915 26488475 PMC4619062

[16] Farooq Z Rocklöv J Wallin J Abiri N Sewe MO Sjödin H Artificial intelligence to predict West Nile virus outbreaks with eco-climatic drivers. Lancet Reg Health Eur. 2022;17:100370. 10.1016/j.lanepe.2022.100370 35373173 PMC8971633

[17] Marini G Manica M Delucchi L Pugliese A Rosà R . Spring temperature shapes West Nile virus transmission in Europe. Acta Trop. 2021;215:105796. 10.1016/j.actatropica.2020.105796 33310078

[18] Reisen WK Fang Y Martinez VM . Effects of temperature on the transmission of west nile virus by Culex tarsalis (Diptera: Culicidae). J Med Entomol. 2006;43(2):309-17. 10.1093/jmedent/43.2.309 16619616

[19] Fornasiero D Mazzucato M Barbujani M Montarsi F Capelli G Mulatti P . Inter-annual variability of the effects of intrinsic and extrinsic drivers affecting West Nile virus vector Culex pipiens population dynamics in northeastern Italy. Parasit Vectors. 2020;13(1):271. 10.1186/s13071-020-04143-w 32471479 PMC7260749

[20] Regional Agency for the Prevention and Environmental Protection of Veneto (ARPAV). Agrometeo Mese N° 6 Maggio 2022. [Weather report, 6 May 2022]. Padova: ARPAV; 2022. Italian. Available from: https://www.arpa.veneto.it/temi-ambientali/agrometeo/file-e-allegati/bollettino-mese/2022/Maggio%202022.pdf

[21] Regional Agency for the Prevention and Environmental Protection of Veneto (ARPAV). Agrometeo Mese N° 7 Primavera 2022. [Weather report, No 7 Spring 2022]. Padova: ARPAV; 2022. Italian. Available from: https://www.arpa.veneto.it/temi-ambientali/agrometeo/file-e-allegati/bollettino-mese/2022/sintesi-2022/Primavera%202022.pdf

[22] Copernicus. Climate bulletins. Brussels: European Commission. [Accessed: 4 Jul 2022]. Available from: https://climate.copernicus.eu/climate-bulletins

